# Electrospun Gelatin–Chondroitin Sulfate Scaffolds Loaded with Platelet Lysate Promote Immature Cardiomyocyte Proliferation

**DOI:** 10.3390/polym10020208

**Published:** 2018-02-21

**Authors:** Francesca Saporito, Giuseppina Sandri, Maria Cristina Bonferoni, Silvia Rossi, Lorenzo Malavasi, Claudia Del Fante, Barbara Vigani, Lauren Black, Franca Ferrari

**Affiliations:** 1 Department of Drug Sciences, University of Pavia, Viale Taramelli 12, 27100 Pavia, Italy; francesca.saporito01@universitadipavia.it (F.S.); cbonferoni@unipv.it (M.C.B.); silvia.rossi@unipv.it (S.R.); barbara.vigani@unipv.it (B.V.); franca.ferrari@unipv.it (F.F.); 2 Department of Chemistry, Physical Chemistry Section, University of Pavia, Viale Taramelli 16, 27100 Pavia, Italy; lorenzo.malavasi@unipv.it; 3 Immunohaematology and Transfusion Service, Apheresis and Cell Therapy Unit, Fondazione IRCCS Policlinico S. Matteo, Viale Golgi 19, Pavia 27100, Italy; c.delfante@smatteo.pv.it; 4 Department of Biomedical Engineering, Tufts University, Medford, MA 02155, USA; lauren.black@tufts.edu

**Keywords:** gelatin and chondroitin sulfate patch, endothelial cells, fetal cardiomyocytes, adhesion and proliferation properties

## Abstract

The aim of the present work was the development of heart patches based on gelatin (G) and chondroitin sulfate (CS) to be used as implants to improve heart recovery after corrective surgery for critical congenital heart defects (CHD). Patches were prepared by means of electrospinning to obtain nanofibrous scaffolds and they were loaded with platelet lysate (PL) as a source of growth factors to further enhance the repair process. Scaffolds were characterized for morphology and mechanical properties and for the capability to support in vitro adhesion and proliferation of dermal fibroblasts in order to assess the system’s general biocompatibility. Adhesion and proliferation of endothelial cells and cardiac cells (cardiomyocytes and cardiac fibroblasts from rat fetuses) onto PL-loaded patches was evaluated. Patches presented good elasticity and high stiffness suitable for in vivo adaptation to heart contraction. CS improved adhesion and proliferation of dermal fibroblasts, as proof of their biocompatibility. Moreover, they enhanced the adhesion and proliferation of endothelial cells, a crucial mediator of cardiac repair. Cell adhesion and proliferation could be related to elastic properties, which could favor cell motility. The presence of platelet lysate and CS was crucial for the adhesion and proliferation of cardiac cells and, in particular, of cardiomyocytes: G/CS scaffold embedded with PL appeared to selectively promote proliferation in cardiomyocytes but not cardiac fibroblasts. In conclusion, G/CS scaffold seems to be a promising system to assist myocardial-repair processes in young patient, preserving cardiomyocyte viability and preventing cardiac fibroblast proliferation, likely reducing subsequent uncontrolled collagen deposition by fibroblasts following repair.

## 1. Introduction

About 1 in every 4 babies born with a heart defect has a critical congenital heart defect (critical CHD, also known as critical congenital heart disease). These are often due to a failure of morphogenesis [1,2] The definitive therapeutic option for critical CHD, such as HLHS (hypoplastic left-heart syndrome) or ToF (tetralogy of Fallot), is a series of corrective surgeries in the first few years of life. These are generally palliative in nature, aimed at maintaining cardiac function as long as possible to increase the likelihood of heart transplant. However, while medical and surgical interventions have significantly improved survival over the years, CHD is still the leading cause of mortality in infants and young children in industrialized countries due to the development of heart failure in many patients [2].

CHD reconstructive surgeries often require surgical placement of implants lacking growth potential [3,4] In fact, current patches consist of synthetic materials (not biodegradable), such as polytetrafluoroethylene (PTFE), which are prone to calcification and require replacement in 14% of patients. In addition, these patches induce an inflammatory foreign-body response, resulting in their encasement in a stiff, fibrous, scar-like tissue without normal cardiac functionality [3,5,6]. As alternatives, allografts, xenografts and autologous tissues such as pericardium and saphenous vein have been used, but they are all associated with similar complications to varying degrees and none have growth potential: graft failure and consequent reoperation are associated with high degrees of morbidity and mortality [3]. As a result, all types of currently available implants increase the risk of infection, hearth failure, arrhythmia and/ or aneurism [5].

In humans, cardiomyocyte proliferation lasts until the heart reaches adult size, and the rate of cardiomyocyte cell cycle activity is highest in infants and declines to very low levels in adults [7,8]. Moreover, although it is not yet fully elucidated which multiple extracellular factors are integrated by signal transduction cascades to control cardiomyocyte proliferation and cardiac regeneration, growth factors produced by epicardium seem to promote the differentiation and proliferation of cardiomyocytes [7,8]. To overcome these limitations, engineered cardiac tissues have been developed in an attempt to restore contractile function to the heart. An optimal bioengineered scaffold for generating engineered myocardial tissue should possess similar properties to those of heart extracellular matrix (ECM), promote cell adhesion and be physiologically reabsorbed as the new tissue regenerates. In addition, it should be able to persist during the regenerative process, in an effort to balance the increasing stiffness of any scar formation [9].

The ideal material for heart repair would be naturally derived, structurally sound, and able to support adhesion and proliferation of cardiomyocytes and endothelial cells while limiting the growth of cardiac fibroblasts, which have the potential to promote scar formation upon surgical healing [3]. Collagen types I and III are the major components of cardiac ECM and glycosaminoglycans can serve as both non-structural and structural proteins [10,11]. Moreover, ECM also has biochemical functions by serving as a reservoir for growth factors [12]. Collagen seems the best candidate to obtain 3D scaffolds for cardiac repair but it has been reported to be an immunogenic material [13,14,15]. On the contrary, gelatin (G), prepared by a denaturation-hydrolysis process starting from natural sources rich in collagen I [16], is described to be non-immunogenic, which appears to be related to the absence of aromatic residues with respect to collagen, as G is deficient in Tyr and Trp and contains only a very small amount of Phe [12].

In this field, one of major challenges is the design of an implant (patch) as a tridimensional (3D) support to mimic cardiac ECM allowing appropriate cell–cell communication and cardiomyocyte contraction. Indeed, 3D nanofiber networks provide a high surface-to-volume ratio and high porosity to promote cell adhesion, proliferation and migration as well as sufficient oxygen and nutrient exchange [17]. Electrospinning is recognized as a simple and efficient enabling nanotechnology to obtain nanofibrous scaffolds [18].

Given these premises, the aim of the present work was the development of heart patches based on gelatin and chondroitin sulfate (CS) to be used as implants to improve heart recovery after corrective surgery for critical CHD. Patches were prepared by means of electrospinning to obtain nanofibrous scaffolds and they were loaded with platelet lysate (PL) as a source of growth factors to further enhance the repair process. CS is a sulfated glycosaminoglycan, which can interact with various positively-charged bioactive molecules and, in particular, with growth factors (GFs) [19,20]. Moreover, it has been described as a proliferation enhancer of corneal cells and dermal fibroblasts [19,20]. Among the different GFs involved in cardiac repair, VEGF (vascular endothelial growth factor), TGF-b1 (transforming growth factor), and FGF (fibroblast growth factor) have a crucial role in promoting angiogenesis, fibrinogenesis and cardiomyocyte proliferation, respectively [7,20]. Moreover in the current literature, there are several reports of CS effectiveness in enhancing cell proliferation in association with PL, a hemoderivative from platelets rich in GF [21,22,23]. This formulative approach was designed to allow the administration of autologous platelet lysate and to assure a fast translation in clinical practice. Patches were characterized for morphology and mechanical properties and for biocompatibility by using normal human dermal fibroblasts (NHDF). Moreover, the capability to support adhesion and proliferation in vitro was assessed by using endothelial cells (HUVEC). Furthermore, patches were embedded with platelet lysate as source of growth factors and the adhesion and proliferation of cardiac cells (cardiomyocytes and cardiac fibroblasts from fetal rats) was evaluated.

## 2. Materials and Methods

### 2.1. Materials

Gelatin (G) from bovine skin 225 g Bloom type B (Sigma Aldrich, Milan, Italy) and chondroitin sulfate 100 EP (CS) low MW 14000 Da, mixture of A (chondroitin 4 sulfate) and C (chondroitin 6 sulfate), (Bioiberica, Barenz, Milan, Italy) were used as polymers.

Platelet lysate (PL) was obtained from the Apheresis Service of the Immunohaematology and Transfusion Service, Apheresis and Cell Therapy Unit (Fondazione IRCCS Policlinico San Matteo, Pavia, Italy), by employing a sterile connection technique. Aliquots of hyperconcentrated platelets (high platelet concentration in small plasma volume and minimal leukocyte contamination) were obtained from apheresis, carried out on regular blood donors. The platelet pool was frozen at −80 °C for 5 h and subsequently thawed in a sterile water bath at 37 °C. An automated platelet count and tests for aerobic, anaerobic and fungal contamination were performed after saline dilution.

### 2.2. Methods

#### 2.2.1. Preparation of Polymeric Solutions

Two different polymeric solutions were prepared: G and G/CS. G was based on gelatin at 20% (w/w) in 10% (w/v) acetic acid, while G/CS was based on gelatin at 20% (w/w) and chondroitin sulfate at 2% (w/w) in 10% acetic acid. This latter solution was prepared by mixing 1:1 in weight ratio of G at 40% (w/w) in 20% w/v acetic acid and CS at 4% (w/w) in distilled water. G solutions were stirred at 40 °C for 1 h. Citric acid (monohydrated citric acid, EP grade, Carlo Erba, Milan, Italy) was added at 5% (w/w) concentration to both G and G/CS solutions.

#### 2.2.2. Rheological Measurements

G and G/CS were subjected to viscosity measurements by means of a rotational rheometer (Rheostress 600, Haake, Enco, Spinea, Italy), equipped with a cone-plate combination (C35/1: diameter = 35 mm; cone angle = 1°) as a measuring system. All measurements were carried out at 25–32–40 °C, after a rest time of 3 min, applying a shear rate of 100 s^−1^. Viscosity values were reported as mean values ± SD (standard deviation) of 3 samples.

#### 2.2.3. Physical Properties of the Solutions

##### Surface Tension

The surface tension of G and G/CS solutions was measured using a tensiometer (DY-300–Kyowa, Niiza-City, Saitama, Japan) having a measurement range between 0 mN·m and 100 mN·m. Measurements were carried out at 25 °C by time-based detection. Surface-tension values were reported as mean values ± SD of 3 samples.

##### Conductivity

The electrical conductivity of G and G/CS solutions was measured by means of an electrical conductometer (FiveGoTM-Mettler Toledo, Milan, Italy), after a calibration with a standard solution having a conductivity of 1413 µs/cm at room temperature. Conductivity values were reported as mean values ± SD of 3 samples.

##### Preparation of Patches (Electrospun Scaffolds)

Scaffolds were obtained by using an electrospinning apparatus (STKIT-40, Linari Engineering, Pisa, Italy), equipped with a high-voltage power supply (Razel R99-E 40, kV), a 10 ml syringe with 21 G needle (0.8 × 20 mm), and a conductive static collector, covered by aluminum foil. Room temperature and humidity were carefully controlled and maintained constant during the electrospinning process at 15% and 25 °C, respectively. A preliminary evaluation of the electrospinning set up was assessed to select optimal conditions to obtain nanofibers without beads. Voltage was tuned from 23 kV to 30 kV, the distance between the collector and the needle from 10 cm to 20 cm, and flux from 0.1 mL/h to 1 mL/h. Based on the quality of each resulting membrane preliminarily evaluated by scanning electron microscopy (SEM), as hereafter described, voltage was fixed at 28 kV, the distance between collector and needle was set to 15 cm, a polymeric solution flux of 0.4 mL/h, and spinning time of 1 h were used, which allowed for the generation of homogeneous membranes. After the preparation procedure, each patch was crosslinked by heating at 150 °C for 2 h.

#### 2.2.4. Patches’ Characterizations

##### Scanning Electron Microscopy (SEM) Analysis

Scaffold morphology was analyzed by means of SEM (Tescan, Mira3XMU, ARVEDI Center, University of Pavia, Pavia, Italy). Samples were sputter-coated by means of graphite deposition under a vacuum. The scaffolds were analyzed before and after heat crosslinking and after 1 week dipped in distilled water. Nanofiber diameters were obtained by means of image analysis software (Image J) from the SEM images. The nanofiber diameters were reported as mean values ± SD of 100 samples.

##### Fourier-Transform Infrared Spectroscopy (FT–IR) Analysis

Spectroscopic measurements were carried out by means of an infrared imaging microscope (Nicolet iN10 MX, Thermo Scientific, ARVEDI Center, University of Pavia, Pavia, Italy). The infrared spectra were acquired over a wavelength range of 4000–500 cm^−1^ at a resolution of 10–20 µm^2^.

##### Mechanical Properties

Scaffolds were subjected to tensile measurements by means of a TA.XT plus apparatus (Stable Microsystems, ENCO, Spinea, Italy), equipped with a 1 kg load cell and A/TG tensile grips. Each scaffold was cut (3 cm length and 1 cm width) and kept vertical by means of two jaws, the lower one fixed and the upper one movable at a constant rate of 0.5 mm/s. Dried or hydrated scaffolds were stretched to fracture and the force as a function of grip distance was recorded. Tensile strength was calculated as the ratio between maximum force recorded (break force) and break area. Elongation percentage was calculated as the percentage ratio between the distance of the two grips at scaffold breaking and the initial scaffold length.

#### 2.2.5. In Vitro Adhesion and Proliferation Assay

##### Normal Human Dermal Fibroblasts (NHDF)

Fibroblasts (normal human dermal fibroblasts, NHDF, from juvenile foreskin, PromoCell, WVR, Milan, Italy) were used between passages 2 and 5 and were cultured in the presence of Dulbecco’s modified Eagle medium (DMEM) (Sigma-Aldrich, Milan, Italy) supplemented with 10% fetal calf serum (FCS, Euroclone, Milan, Italy), 200 IU/mL penicillin and with 0.2 mg/mL streptomycin (Sigma-Aldrich, Milan, Italy) and kept at 37 °C in a 5% CO_2_ atmosphere with 95% relative humidity (RH).

##### Human Umbilical Vein Endothelial Cells (HUVEC)

Endothelial cells from human umbilical vein (HUVEC) (Lonza, Milan, Italy) were used between passages 2 and 5 and were cultured in endothelial basal medium (EBM2) (Lonza, Milan, Italy) supplemented with 2% FCS, 0.4% hFGF-B, 0.1% VEGF, 0.1% R3-IGF-1, 0.1% ascorbic acid, 0.1% hEGF, 0.1% heparin and 0.04% hydrocortisone (Lonza, Milan, Italy) and with 1% penicillin/streptomycin (Sigma-Aldrich, Milan, Italy), and kept at 37 °C in a 5% CO_2_ atmosphere with 95% RH.

##### Adhesion and Proliferation Tests

Patches were cut to have a final area of 0.36 cm^2^ (0.2 cm diameter) to cover the bottom of a 96-well plate. Both fibroblasts and endothelial cells were seeded onto each scaffold at 10^5^ cells/cm^2^ seeding density and grown for 7 days. Fibroblast growth on tissue-culture plastic and endothelial growth on collagen-coated tissue-culture plastic were considered as standard growth (GM—growth medium).

At prefixed end points, cell growth was assessed by means of a MTT test, SEM analysis or confocal-laser scanning microscopy (CLSM) analysis after nuclear and cytoskeleton staining.

##### MTT Test

50 µl of MTT solution (Sigma-Aldrich, Milan, Italy) at 2.5 mg/mL concentration in HBSS (Hank’s buffered salt solution) pH 7.4 was added to cover each scaffold for 3 h. 22 Subsequently, MTT solution was removed from each well, and the substrates were washed with 200 µL of phosphate-buffered saline (PBS). After the removal of PBS, 100 µL of dimethyl sulfoxide (DMSO) was put in each well, and the absorbance was assayed at 570 nm by means of an enzyme-linked immunosorbent assay (ELISA) plate reader (Imark Absorbance Reader, Biorad, Segrate, Italy), with a reference wavelength of 690 nm. Cell viability was expressed as optical density (OD).

##### SEM Analysis

Cell substrates grown onto scaffolds were fixed using a 3% (w/v) glutaraldehyde solution in PBS (Sigma-Aldrich, Milan, Italy) for 2 h at room temperature. Then, the substrates were washed three times with PBS and dehydrated by means of ethanol solutions at increasing concentrations (50–75–100% v/v). Scaffolds were sputtered with platinum and analyzed as previously described above.

##### Confocal-Laser Scanning Microscopy (CLSM) Analysis

Cells grown on the scaffolds were fixed using a 3% (w/v) glutaraldehyde solution in PBS (Sigma-Aldrich, Milan, Italy) for 2 h at room temperature. The substrates were than washed three times with PBS. Cellular cytoskeletons were stained by incubating with 50 µl (50 µg/ml) phalloindin-TRITC (Sigma-Aldrich, Milan, Italy) for 40 min, in the dark. Then each substrate was washed twice and cell nuclei were stained with 100 µL of Hoechst 33258, diluted 1:10000 (Sigma-Aldrich, Italy), for 10 min in the dark.

Scaffolds were placed on a microscope slide and imaged using a CLSM (Leica TCS SP2, Leica Microsystems, Milan, Italy) with λex = 346 nm and λem = 460 nm for Hoechst 33258 and λex = 540 nm and λem = 570 nm for phalloindin-TRITC. The acquired images were processed with software associated with the microscope (Leica Microsystem, Milan, Italy).

#### 2.2.6. In Vitro Adhesion and Proliferation Assay: Cardiac Cells

Cardiac cells were obtained from rat heart fetuses. All animal procedures were performed in accordance with the standard international ethical guidelines (European Communities Council Directive 86/609/EEC) approved by Italian Health Ministry (D.L. 116/92). The study protocol was approved by the Local Institutional Ethics Committee of the University of Pavia for the use of animals.

Adult female Sprague Dawley rats (Sprague Dawley SD) of 17–18 days gestation were anesthetized with intramuscular injection of Zoletil^®^ 50/50 mg/mL (250 mg tiletamine in powder (as hydrochloride) and 250 mg zolazepam in powder (as hydrochloride) per mL, (Virbac S.r.l., Milan, Italy)) at 1 mL/kg dose. After cervical dislocation of the mother rat, fetuses were euthanized by conscious decapitation and their hearts were collected in ice-cold sterile PBS supplemented with 20 mM glucose and 1 mL/L of penicillin–streptomycin solution (100 IU/mL and 100 μg/mL respectively, Sigma-Aldrich, Milan, Italy).

Heart tissues were transferred to a conical tube and 7 mL of collagenase solution (15000 IU/mL collagenase from *Clostridium histolyticum* (Sigma-Aldrich, Milan, Italy) were added and the tissue was digested for 7 min with stirring on the orbital shaker. Hearts were then triturated 5 times in a 5 mL pipette and the larger chunks were allowed to settle to the bottom of the tube before the supernatant was collected. The undigested tissues were subjected to subsequent digestion cycles until complete digestion of the tissue. The supernatants were filtered (70 µm pore size membrane, WVR, Milan, Italy) and the resulting cells were centrifuged at 500 rpm for 10 min, resuspended in DMEM containing 10% w/w horse serum (Sigma-Aldrich, Milan, Italy), 2% w/w FBS and 1% w/w pen/strep/ampho (Sigma-Aldrich, Milan, Italy) and seeded at a density of 10^5^ cells/cm^2^ onto each scaffold (0.36 cm^2^–0.2 cm diameter) in a 96 well-plate. Scaffolds were employed as such or embedded with 100 μL of platelet lysate. Cells were grown for 3 days. Cardiac cells grown directly onto tissue culture plastic in standard growth condition (GM) or in presence of PL (PL—growth medium supplemented with PL in the same amount as in presence of scaffolds) were considered as control conditions (GM and PL, respectively).

At prefixed end points, cell growth was assessed by means of a MTT test, SEM analysis (both as previously described), or CLSM analysis after staining, as described below.

##### CLSM Analysis

Cells grown on scaffolds were fixed using a 3% (w/v) glutaraldehyde solution in PBS (Sigma-Aldrich, Milan, Italy) for 2 h at room temperature. The substrates were than washed three times with PBS. Cardiomyocytes were stained by using 50 µL anti-α-actinin (sarcomeric) antibody produced in mouse (30.3 mg/mL diluted at 1:1600, Sigma-Aldrich, Milan, Italy) (contact time 1 h), and after washing with PBS primary antibodies were conjugated with 50 µL fluorescence secondary antibody CF™ 555 (TRITC, λex = 555 nm, λem = 565 nm) (2 mg/mL concentration diluted at 1:400, Sigma-Aldrich, Milan, Italy) (contact time 1 h).

Cardiac fibroblasts were stained by using 50 µL anti-actin α-smooth muscle antibody (mouse monoclonal) produced in mouse (2.3 mg/mL diluted at 1:460, Sigma-Aldrich, Milan, Italy), and after washing with PBS primary antibodies were conjugated with 50 µL the secondary antibody CF™ 555 (TRITC, λex = 555 nm, λem = 565 nm).

Subsequently, all the substrates were assessed for cell proliferation by staining with 50 µL Ki-67 primary antibody (Sigma-Aldrich, Milan, I), and, after washing with PBS, primary antibodies were conjugated with the fluorescence secondary antibody Atto 488 goat anti-rabbit IgG (Atto 488, λabs = 501 nm, λem = 523 nm) (1 mg/mL concentration diluted at 1:200, Sigma-Aldrich, Milan, Italy).

All cardiac cell nuclei were stained with Hoechst 33258, as previously described.

As above, scaffolds were placed on a microscope slide and imaged using a CLSM, as previously described.

#### 2.2.7. Statistical Analysis

Statistical differences were evaluated by means of t-test, (Statgraphics Centurion XV, Statistical Graphics Corporation, The Plains, Virginia, USA). Differences were considered significant at p < 0.05 and each p value is reported in the text or in the captions.

## 3. Results

### 3.1. Characterization of Polymeric Solutions

#### 3.1.1. Rheological Properties

Viscosity is a key factor in order to obtain continuous and homogeneous fibers. Acetic acid was previously described as a fundamental component to allow gelatin spinnability as it avoids gelatin gelation [24]. Moreover, by increasing the acetic acid concentration the surface tension decreases [25]. Optimal viscosity of gelatin solutions to obtain reproducible fibers was characterized in the range of 200–1500 mPa·s at 25 °C [24]. Gelatin entanglement concentration was previously described as higher than 14% w/w [26]. The viscosity of G and G/CS polymeric solutions was characterized at 25, 32 and 40 °C at 100 s^−1^ shear rate. G presented a viscosity of 458 ± 11, 342 ± 4 and 323 ± 25 mPa·s at 25, 32 and 40 °C, respectively (t-test: 25 °C vs 32 °C: p < 0.001; 25 °C vs 40 °C: p = 0.003; 25 °C vs 40 °C: p = 0.375), while G/CS had a viscosity of 200 ± 5, 117 ± 7 and 82 ± 6 mPa·s at 25, 32 and 40 °C, respectively (t-test: 25 °C vs 32 °C: p < 0.001; 25 °C vs 40 °C: p < 0.001; 32 °C vs 40 °C: p = 0.003). The viscosity of both G and G/CS solutions decreased by increasing temperature, confirming that the presence of acetic acid impaired gelation, favoring spinnability. At each temperature, the G solution showed higher viscosity values than the G/CS solution (t-test: G vs G/CS at 25 °C: p < 0.001; 32 °C: p < 0.001; 40 °C: p < 0.001). The decrease of viscosity due to the addition of CS to G was probably due to a charge–charge interaction between G and CS chains and the resulting interaction product was probably characterized by a higher degree of chain entanglement resulting in a decrease of consistency. Gelatin and chondroitin sulfate interaction was previously reported in a paper by Chang [23] describing hydroxyapatite gelatin nanocomposite modified with the addition of chondroitin sulfate.

#### 3.1.2. Conductivity and Surface Tension

During the electrospinning process, in the needle tip (electrode), the generation of charges within the fluid to be electrospun allows the repulsion between charges at the free surface that works against surface tension and fluid elasticity to deform a droplet into a conical shape called a Taylor cone. The cone is unstable and a jet of fluid is emitted from the needle tip to form a filament that is accelerated and stretched due to the electric field and collected upon the electrode plate [27]. To allow the electrospinning process to work effectively, conductivity should be at least of 100 µS/cm [27] and surface tension should range from 50–30 mN/m [25]. G solution had an electrical conductivity of 2585 ± 15.3 µS/cm and a surface tension of 40 ± 1.0 mN/m while G/CS had an electrical conductivity of 3450 ± 11.8 µS/cm and a surface tension of 41 ± 0.1 mN/m (t-test: conductivity: G vs G/CS: p < 0.001; surface tension: G vs G/CS: p = 0.121). While the surface tension was similar in the two solutions, G/CS possessed conductivity significantly higher than that of G/CS. This is likely related to the higher charge density of the polymeric mixture due to the presence of CS, which has a maximum charge density of 1, as there are a number of negatively charged functional groups per repeated unit.

CS did not alter G solution properties, and 0.5% w/w was allowed to maintain conductivity and surface tension suitable for the electrospinning process.

### 3.2. Characterization of Nanofibrous Scaffolds

**Figure 1:**
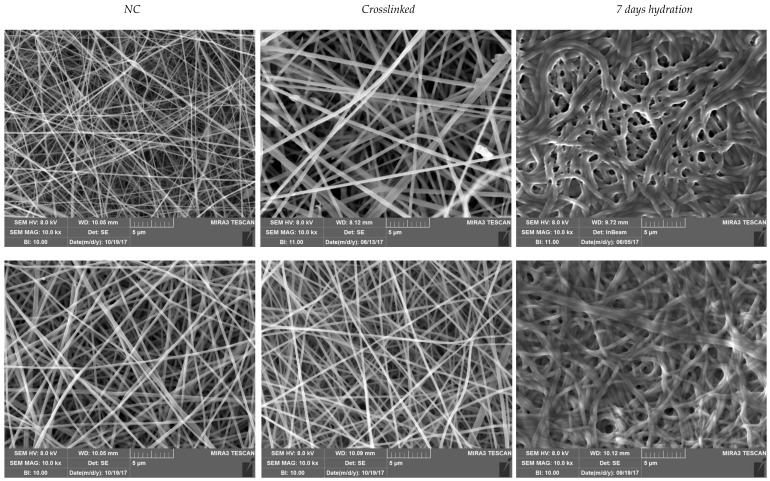
Scanning electron microscopy (SEM) microphotographs of nanofibrous scaffolds immediately after preparation (not crosslinked, NC), after cross-linking, and after 6 days hydration in water.

SEM microphotographs of nanofibrous scaffolds immediately after preparation, after cross-linking and after 6 days in water are shown in Figure 1. G and G/CS nanofibers showed a random orientation with regular structure and a smooth surface, without the presence of beads or ribbons. The cross-linking did not change fiber morphology. After 6-days of hydration, the nanofibrous structure was still present although the inter-fiber spaces (porosity) were smaller.

Nanofiber diameters were measured immediately after preparation (not crosslinked, NC), after cross-linking, and after 6 days in water. G scaffolds had 151 (±0.03) nm fibers and the cross-linking caused a slight increase in diameters to 180 (±0.03) nm without any visible alteration of system morphology. G/CS scaffolds had smaller fiber diameters of 120 nm (±0.09) in comparison with G scaffolds and again the crosslinking did not markedly modify the fiber dimensions (109 ± 0.02). G scaffolds and G/CS scaffolds were characterized by not dramatically different fiber dimensions indicating that the addition of chondroitin sulfate to gelatin did not alter spinning process and fiber formation (t-test: G scaffold: NC vs crosslinked: p = 0.087; NC vs hydrated: p < 0.001; crosslinked vs hydrated: p < 0.001; G/CS scaffold: NC vs crosslinked: p = 0.252; NC vs hydrated: p < 0.001; crosslinked vs hydrated: p < 0.001; NC: G vs G/CS: p < 0.001; crosslinked: G vs G/CS: p = 0.006; hydrated: G vs G/CS: p < 0.001). On the other hand, 6 days hydration caused a significant increase of nanofiber diameters for both the nanofibrous scaffolds: hydrated G scaffolds had nanofiber diameters of 446 (±0.10) nm while hydrated G/CS scaffolds had diameters of 306 (±0.06) nm. The presence of chondroitin sulfate led to a significantly lower degree of swelling, which could be related to the presence of interactions between gelatin and chondroitin sulfate interactions [28]. The hydration conceivably allowed nanofiber swelling without modification of nanofibrous structure. The correlation between fiber dimensions (diameter) and cell adhesion and proliferation is not completely understood: structural similarity of the fibers to the native ECM and physicochemical characteristics of the polymer-forming fibers seem crucial, moreover topographical surface (fibers and pores) seem fundamental for cell migration [29].

**Figure 2:**
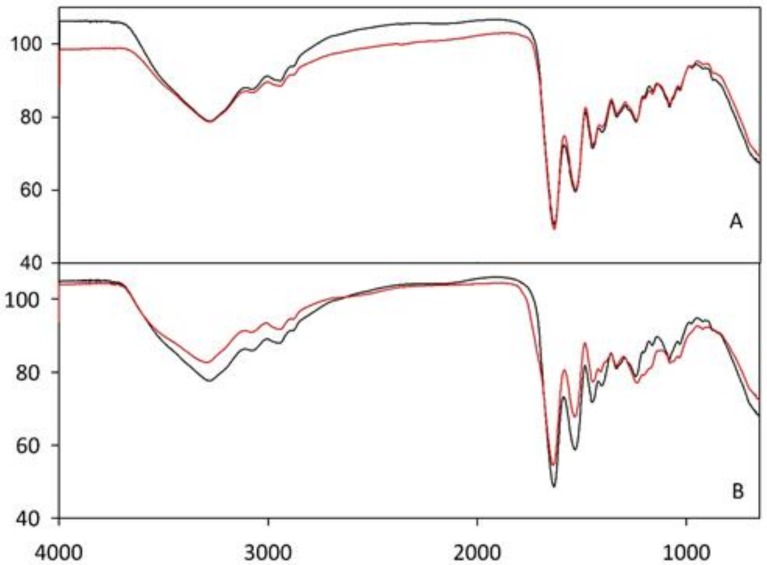
Fourier–transform infrared (FT–IR) spectra for G (A) and G/CS (B) scaffolds before (black line) and after crosslinking (red line).

Figure 2 reports Fourier-transform infrared (FT–IR) spectra for G (A) and G/CS (B) scaffolds, comparing the profiles before and after crosslinking. For the G scaffold, the two spectra were superimposed thus indicating the absence of any significant modification due to the crosslinking. For the G/CS scaffold, small variations were found in the region below ca. 1200 cm^−1^. This region of the FT–IR spectra is of complex origin and considered of limited use for the extraction of structural information [30]. However, it has been associated with a possible disorder in gelatin molecules and more likely associated with the loss of the triple helical state.

The absence of any variations at higher wavenumbers, in particular in the region defined as amide A at around 3400 cm^−1^ and amide B at around 3090 cm^−1^, confirms the absence of any newly formed hydrogen bonds or any variations in the backbone conformation or hydrogen bonding pattern [31].

The combination of gelatin and chondroitin sulfate was previously used to develop a sponge-like scaffold for corneal stromal tissue engineering [32]. However the chemical cross-linking of gelatin and the chemical grafting of chondroitin sulfate onto gelatin structure caused the formation of new covalent bonds, deeply changing the chemical identity of each polymeric material. Moreover, in the context of a potential fast translation into clinical practice, the absence of new chemical bond formation in G/CS scaffolds suggested by FT–IR analysis could be a great advantage from the regulatory point of view.

**Figure 3:**
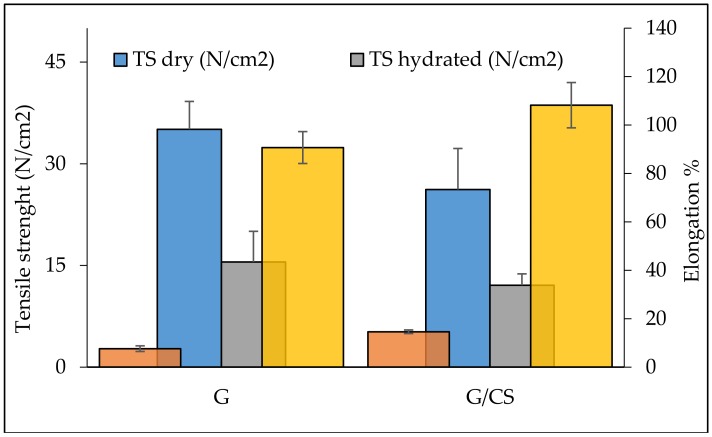
Scaffold mechanical properties as tensile strength (TS) (N/cm^2^) and elongation % measured for dry and hydrated scaffolds (mean values ± SD; n = 20). (red lines: Max: maximum tensile strength during systole; Min: minimum tensile strength during diastole) (t-test: tensile strength: dry: G vs G/CS: p = 0.218; hydrated: G vs G/CS: p = 0.693; G: dry vs hydrated: p = 0.003; G/CS: dry vs hydrated: p = 0.105; elongation: dry: G vs G/CS: p = 0.0.36; hydrated: G vs G/CS: p = 0.179; G: dry vs hydrated: p < 0.001; G/CS: dry vs hydrated: p < 0.001).

Figure 3 reports the results of scaffold mechanical properties as tensile strength (N/cm^2^) and elongation % measured for dry and hydrated scaffolds. Overall, tensile strength was higher when the scaffolds were in the dry state while the G scaffold was characterized by a significantly higher force at breaking compared to the G/CS scaffolds. In the hydrated state, G scaffolds maintained a stiffness greater than that of the G/CS scaffolds, although both scaffolds presented significantly lower tensile strength than in the dry state. On the contrary, elongation (directly related to scaffold elasticity) was significantly higher in the hydrated state for both the systems, and G/CS had an elasticity greater than that of G scaffold. The presence of CS in the nanofiber structure could change the gelatin tridimensional conformation, causing a decrease in chain organization, which could eventually cause a partial loss of stiffness. Since myocardial tissue is characterized by left ventricle stiffness ranging from 1–2 N/cm^2^ (10–20 kPa) during diastole and 20–50 N/cm^2^ (200–500 kPa) during systole [33] and, additionally, knowing that cardiomyocytes function best if the scaffold mimics the native tissue’s mechanical properties [34,35], optimal heart patches should have a stiffness in the range of several tens of kPa (starting from 1 N/cm^2^) to about 1 MPa (up to 100 N/cm^2^), based on theoretical simulations [33]. Considering this, and considering that the patches were designed to be put in contact with heart walls without enveloping the whole hearth, G and G/CS scaffolds showed mechanical properties that should be suitable for in vivo application.

### 3.3. In Vitro Adhesion and Proliferation Assay: Fibroblasts and Endothelial Cells

**Figure 4:**
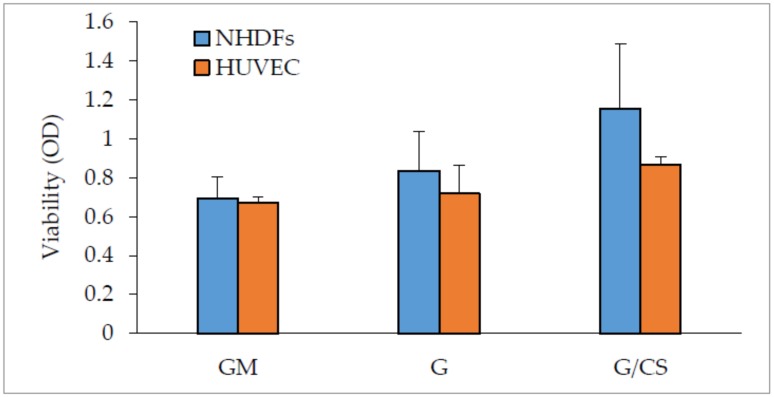
Normal human dermal fibroblasts (NHDFs) and human umbilical vein endothelial cells (HUVEC) viability (optical density, OD) evaluated for cells grown onto G and G/CS scaffolds and in standard conditions (GM—growth medium), for 7 days; cells grown directly on plastic well bottom was considered as standard growth (GM) (mean values ± SD; n = 8) (t-test: NHDF: GM vs G: p = 0.107; GM vs G/CS: p = 0.002; G vs G/CS: p = 0.035; HUVEC: GM vs G: p = 0.388; GM vs G/CS: p < 0.001; G vs G/CS: p = 0.014; GM: NHDF vs HUVEC: p = 0.579; G: NHDF vs HUVEC: p = 0.200; G/CS: NHDF vs HUVEC: p = 0.028).

Figure 4 reports fibroblast and endothelial cell (HUVEC) viability (optical density, OD) evaluated for cells grown on G and G/CS scaffolds in standard conditions (GM), for 7 days.

Dermal fibroblasts were chosen to assess the biocompatibility of the patches [36,37] and, moreover, to understand if the nanofibrous structure of the scaffolds was able to act as a support for cardiac cells by simulating extracellular matrix architecture.

Based on the NHDF data, fibroblast adhesion and growth on the G scaffolds was not significantly different with respect to standard growth (GM) conditions.

G/CS scaffolds were amenable to fibroblast adhesion and growth to a significantly greater extent as compared to standard growth (GM) conditions, indicating that this scaffold was likely able to enhance cell adhesion and proliferation. The capability of G and G/CS patches to act as scaffolds and to support fibroblast adhesion and proliferation was proof of their biocompatibility.

Endothelial cells have a crucial role in maintaining vascular homeostasis to restore endothelial integrity and to participate in new blood-vessel formation. Moreover, endothelial cells have been described as the main players in releasing growth factors, cytokines and chemokines in specific conditions such as hypoxia and shear stress [38].

Based on the endothelial cell (HUVEC) data, cell adhesion and growth was not significantly different on the G scaffolds as compared to standard growth (GM) conditions.

G/CS scaffolds enhanced the adhesion and growth of endothelial cells to a significantly greater extent as compared to standard growth (GM) conditions, indicating that this scaffold was also able to enhance HUVEC adhesion and proliferation.

Chondroitin sulfate seems to have a fundamental role in promoting HUVEC proliferation: in fact it was previously described as an enhancer of cell adhesion and proliferation of both fibroblasts and endothelial cells [20].

**Figure 5:**
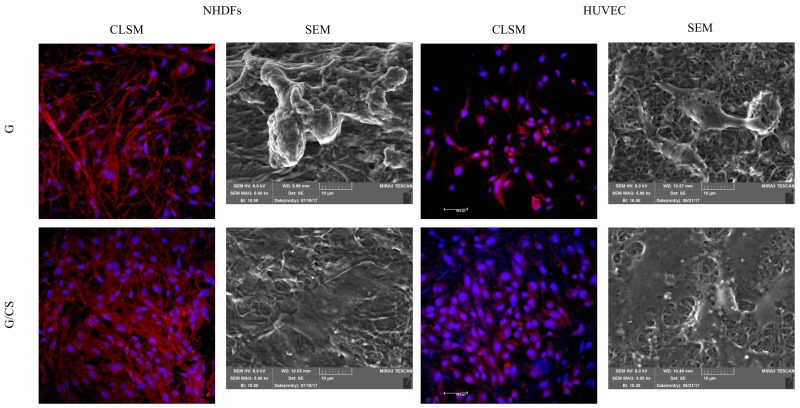
Microphotographs of NHDFs and HUVEC grown on G and G/CS scaffolds for 7 days: confocal-laser scanning microscopy (CLSM) (nuclei in blue—Hoechst 33258; cytoskeleton in red, TRICT-phalloidin) and SEM analysis.

Figure 5 reports microphotographs of fibroblasts and HUVECs grown on G and G/CS scaffolds for 7 days. The cell substrates were subjected to CLSM (staining nuclei in blue—Hoechst 33258; cytoskeleton in red—TRICT-phalloidin) and SEM analysis.

CLSM analysis confirmed viability results. Independently of the scaffold type, fibroblasts maintained their fusiform shape, as demonstrated by cytoskeleton labelling (red staining), and normal nuclei (blue staining). Moreover, it is clear that G/CS scaffolds enhanced cell growth and proliferation better than the G scaffold as cells reached subconfluence.

In addition, endothelial cells (HUVEC) maintained normal nuclei (blue staining) and cytoskeletons (red staining) and showed their classical polygonal shape. Moreover, G/CS scaffolds again led to cell confluence, likely because of enhanced cell growth and proliferation as compared to the G scaffold.

SEM imaging demonstrated that the cells adhered to the nanofibrous scaffolds and that they appeared fully integrated and in contact with them. G scaffolds allowed fibroblast or endothelial cell growth in isolated clusters localized on nanofibers or as agglomerated groups adhered to the nanofiber scaffold surface, and both cell types demonstrated a relatively smooth surface with some granules. On the contrary, both fibroblasts and endothelial cells formed a uniform sheet on G/CS scaffolds showing a relatively homogeneous surface with some granules as well. Moreover, in the case of the G/CS scaffolds, the nanofibers were well integrated in the biological substrate. Endothelial cell proliferation is a crucial mediator of cardiac repair [39]. In fact, heart muscle, and in particular cardiomyocytes, have a high metabolic rate, which is reflected by high capillary density in the heart. It should come as no surprise that vascularization is one of the greatest challenges in cardiac-tissue engineering [40], and thus a scaffold that promotes endothelial cell growth and attachment is desirable, as in this case.

### 3.4. In Vitro Adhesion and Proliferation Assay: Cardiac Cells

**Figure 6:**
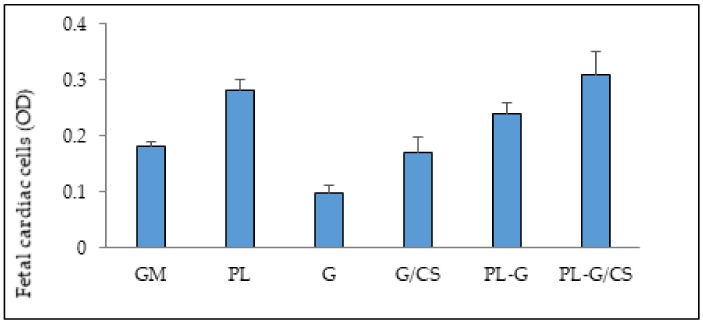
viability (optical density, OD) of cardiac cells (cardiomyocytes and cardiac fibroblasts) evaluated for cells grown onto G and G/CS scaffolds and in standard conditions (GM), for 3 days without or in presence of platelet lysate (PL-G and PL-G/CS); cardiac cells grown directly onto tissue culture plastic in standard growth condition (GM–growth medium) or in the presence of PL (PL–growth medium supplemented with PL in the same amount as in presence of scaffolds) were considered as control conditions (GM and PL, respectively) (mean values ± SD; n = 8) (t-test: w/o PL: GM vs G: p < 0.001; GM vs G/CS: p = 0.654; G vs G/CS: p = 0.027; with PL: GM vs PL: p = 0.002; GM vs G: p = 0.025; GM vs G/CS: p = 0.012; PL vs G: p = 0.152; PL vs G/CS: p = 0.561; G vs G/CS: p = 0.153; G: with PL vs w/o PL: p < 0.001; G/CS with PL vs w/o PL: p = 0.009).

Figure 6 shows the viability (optical density, OD) of cardiac cells (a mixed population of cardiomyocytes and cardiac fibroblasts isolated from the heart) evaluated for cells grown on the G and G/CS scaffolds and in standard conditions (GM), for 3 days with or without the addition of platelet lysate (PL or w/o PL, respectively). Cardiac cells grown directly on tissue culture plastic with or without PL were considered control conditions (PL and GM, respectively).

PL was extemporaneously absorbed onto scaffolds. This procedure was designed to allow the loading of autologous platelet lysate and to avoid a possible risk related to hemoderivatives from donors. Moreover, there is evidence that autologous platelet lysate should favor the repair process [41]. Cardiac cells adhered and proliferated on G/CS scaffold similar to standard conditions (GM). On the contrary, PL-embedded scaffolds demonstrated increased cell proliferation with respect to standard conditions (GM), which was significantly higher for the G/CS scaffold, although it was not significantly different from cardiac-cell proliferation due to PL alone. The presence of PL significantly increased cardiac-cell adhesion and proliferation, and chondroitin sulfate appears to have a synergic effect in promoting cardiac cell growth.

**Figure 7:**
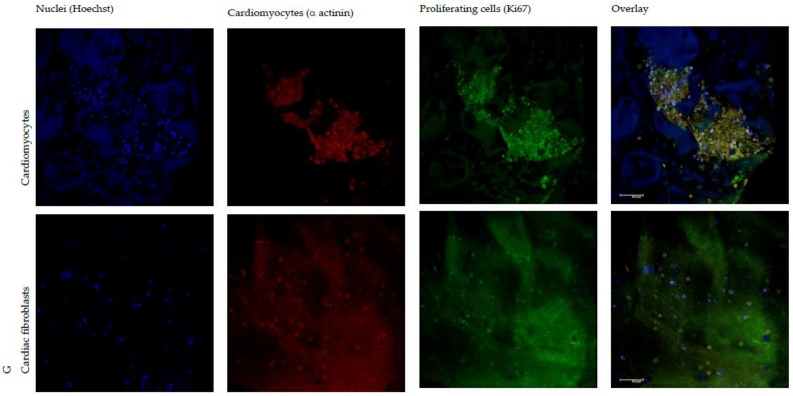

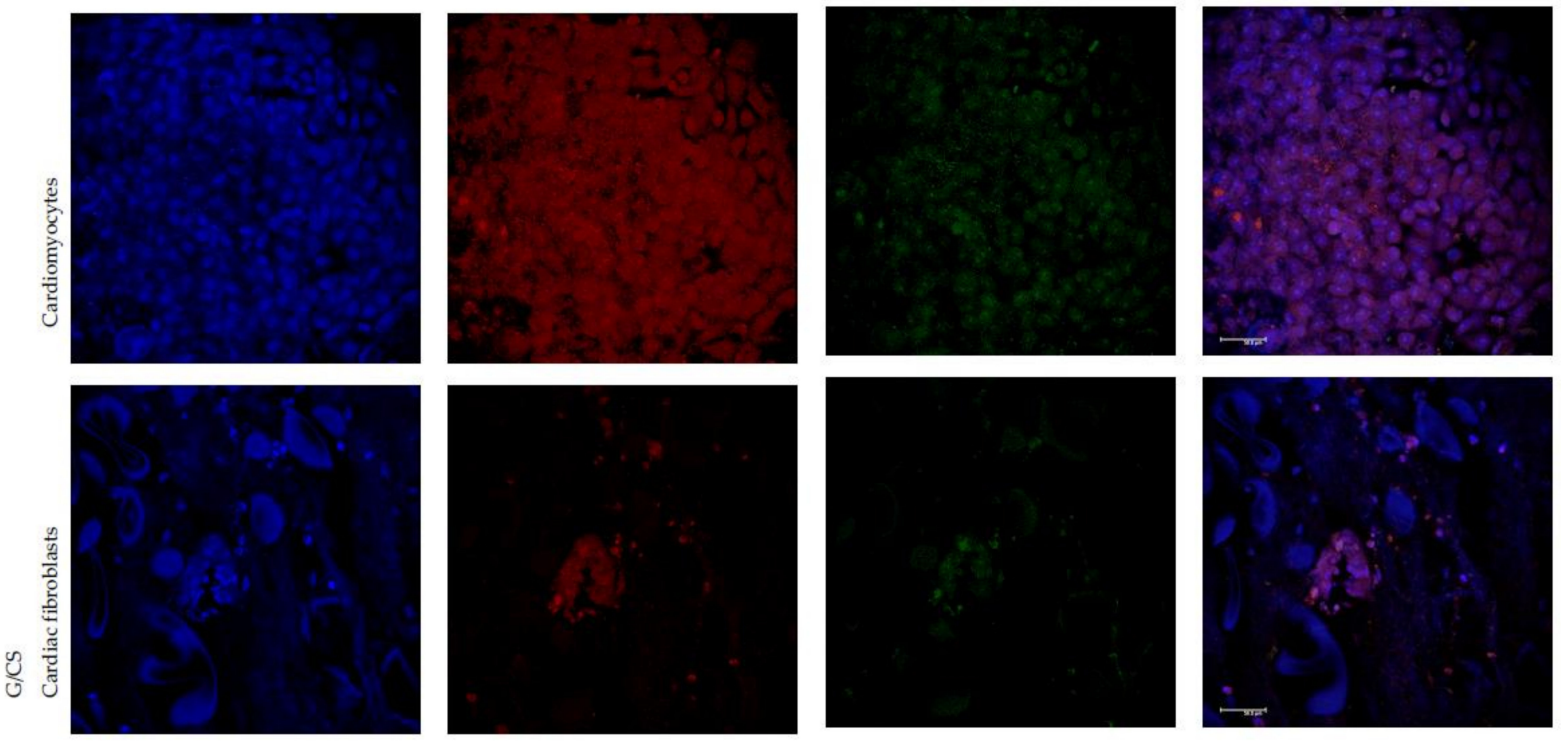
CLSM microphotographs of cardiomyocytes grown onto G or G/CS scaffolds in the presence of platelet lysate (nuclei in blue-Hoechst 33258; muscle fiber in red − α actinin antibody; proliferation marker in green–KI67).

Figure 7 shows CLSM microphotographs of cardiac cells (cardiomyocytes and cardiac fibroblasts, respectively) grown on G or G/CS scaffolds in the presence of platelet lysate (nuclei in blue-Hoechst 33258; muscle fiber in red − α actinin or smooth muscle actin antibodies; proliferation marker in green–KI67). In the presence of PL, the proliferation on G/CS scaffolds led cardiomyocytes to grow to confluence, while cardiomyocytes grown on the G scaffolds did not reach confluency and cells were isolated and not in contact with each other. Furthermore, cardiac fibroblasts (also present in the mixed-cell isolate from hearts) adhered and proliferated to a lesser extent in comparison with cardiomyocytes in both G and G/CS scaffolds. Even if the proliferation results (Figure 6) demonstrated that cell growth onto PL G and PL G/CS scaffolds was similar, the prevalence of cardiomyocyte proliferation was higher for G/CS scaffolds. These results suggest that the G/CS scaffold embedded with PL seems to favor cardiomyocytes’ proliferation rather than cardiac-fibroblast proliferation, which is an important finding for future in vivo applications where cardiomyocyte growth should be promoted and cardiac-fibroblast proliferation limited [39].

**Figure 8:**
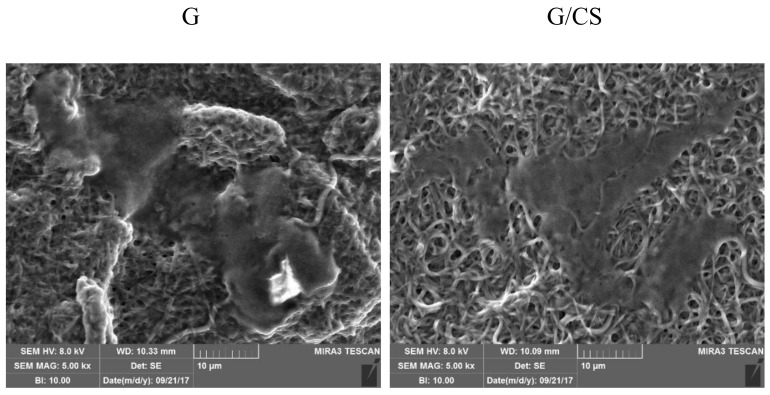
SEM microphotographs of G and G/CS scaffolds embedded with platelet lysate after 3 days of culture of cardiac cells.

Figure 8 reports SEM microphotographs of G and G/CS scaffolds embedded with platelet lysate after 3 days of culture of cardiac cells. G and G/CS scaffolds allowed the adhesion and the proliferation of cardiac cells (both cardiomyocytes and fibroblasts) to form a uniform sheet with a homogeneous surface. The deep interconnection between cell substrate (at confluency) and the nanofibrous structure with an inclusion of the fibers into the biological matrix is visible.

## 4. Conclusions

G and G/CS patches were prepared by means of electrospinning and were cross-linked by heating in the presence of citric acid. Nanofiber structures were not modified by the cross-liking process or by hydrating in water. Scaffolds presented good elongation (elasticity) combined with high stiffness; and the presence of CS in the nanofiber structure improved mechanical properties (lower stiffness and higher elasticity), probably due to a change in the gelatin tridimensional conformation and a possible decrease in chain organization, making it more suitable for in vivo adaptation to heart contraction. In addition, the presence of CS improved the adhesion and proliferation of dermal fibroblasts, which can be considered as proof of biocompatibility. Moreover, developed patches were able to enhance the adhesion and proliferation of endothelial cells, which are crucial mediators of cardiac repair. The capability of patches to act as scaffolds to cell adhesion and growth could be related to their elastic properties, which could favor cell motility.

The extemporaneous loading of platelet lysate by embedding patches seems to further enhance the adhesion and proliferation of cardiac cells and, in particular, of cardiomyocytes. This loading strategy could be easily adapted to employ autologous platelet lysate. Furthermore, the presence of CS had a synergic effect with PL: the G/CS patch appears to selectively favor the proliferation of cardiomyocytes rather than cardiac fibroblasts.

In conclusion, this G/CS scaffold system seems promising in assisting myocardial repair due to a double beneficial effect: first, it has a direct effect on enhancing endothelial-cell proliferation and cardiomyocyte proliferation; and, second, it should control fibrosis due to a reduction in cardiac-fibroblast proliferation. This formulative approach was designed to facilitate a fast translation into clinical practice.
